# Clinical outcomes of preimplantation genetic testing for structural rearrangements in couples with chromosomal inversions: a retrospective analysis

**DOI:** 10.3389/fgene.2026.1779551

**Published:** 2026-04-24

**Authors:** Yutong Li, Yuezhi Keqie, Cuiting Peng, Han Chen, Jun Ren, Hong Yang, Zhushu Liu, Wei Fan, Shan Luo, Xuemei Zhang, Ting Hu, Shanling Liu, Xinlian Chen, Fan Zhou

**Affiliations:** 1 Department of Medical Genetics Center, West China Second University Hospital, Sichuan University, Chengdu, Sichuan, China; 2 Key Laboratory of Birth Defects and Related Diseases of Women and Children (Sichuan University), Ministry of Education, Chengdu, Sichuan, China; 3 Department of Reproductive Medicine, West China Second University Hospital, Sichuan University, Chengdu, Sichuan, China

**Keywords:** aneuploidy, blastocysts, chromosomal inversion, genetic counseling, preimplantation genetic testing

## Abstract

Chromosomal inversion is one of the common types of chromosomal structural rearrangements. For couples with chromosomal inversions, the appropriate recommendations for preimplantation genetic testing (PGT) remain a subject of ongoing debate. This study retrospectively included couples who underwent PGT between January 2019 and December 2024. All included couples were classified into subgroups based on karyotyping analysis: PGT-INV (chromosomal inversion), PGT-PV (chromosomal polymorphic variation), PGT-A (normal karyotype), and PGT-SR (other balanced chromosomal rearrangements). The euploid rate of biopsied blastocysts did not differ significantly among the PGT-INV, PGT-PV, and PGT-A groups (*P* > 0.05); however, a statistically significant difference was observed between the PGT-SR group and each of the other three groups (*P* < 0.001). The aneuploidy rate in couples with female inversion was significantly higher than that in couples with male inversion (*P* = 0.046). Meanwhile, the proportion of aneuploid blastocysts associated with rearrangement was significantly higher in pericentric (38.94%) compared to paracentric inversions (23.60%) (*P* = 0.022). With the inverted fragment size increased, the proportion of aneuploid blastocysts associated with rearranged chromosomes increased accordingly and exhibited a linear trend (*P* < 0.05). In conclusion, the overall euploid rate of blastocysts in couples with chromosomal inversion showed no statistically significant difference compared to those with normal karyotype and chromosomal polymorphic variation; however, the carrier gender and the size of the inverted fragment are influencing factors for the abnormality rate associated with homologous rearranged chromosomes. Genetic counseling is strongly recommended for couples carrying chromosomal inversions.

## Introduction

Chromosomal inversions arise when a chromosomal segment experiences two breaks and is subsequently reinserted in a reversed direction. Inversion constitutes one of the three common types of balanced chromosomal rearrangements (Kaiser, 1984; Fryns and Van den Berghe, 1980). The incidence of chromosomal inversions has been estimated at 0.12% based on a study of 8,158 consecutive prenatal genetic testing samples (Van Dyke et al., 1983), and reported to be 0.229–0.344% in couples with recurrent fetal wastage (Fryns and Van Buggenhout, 1998). Although individuals with chromosomal inversions usually have no obvious loss of genetic material and present with a normal phenotype. These couples may encounter reproductive issues, including infertility resulting from disrupted spermatogenesis, repeated pregnancy losses, or an increased likelihood of having children with congenital malformations. Carriers of pericentric inversions have been found to face an estimated 5%–10% chance of producing offspring with an unbalanced karyotype, according to previous reports (Tong et al., 2022).

Depending on whether the involved chromosomal fragment contains a centromere, inversions are classified as pericentric or paracentric. Pericentric inversions involve inverted segments spanning both the long and short arms of the chromosome, which include the centromere. Whereas, paracentric inversions involve a segment located entirely within either the long or short arm and do not include the centromere. During primordial germ cell meiosis, the formation of an inversion loop results from homologous pairing, which facilitates crossing-over and can lead to gamete imbalances, including partial monosomy, partial trisomy, and acentric or dicentric chromosomes through aberrant recombination (Kaiser, 1984; Fryns and Van den Berghe, 1980). Importantly, whether an inversion loop is formed is largely determined by the size of the inverted segment. As larger segments increase the probability of meiotic imbalances and adverse pregnancy outcomes. Evidence suggests that an inverted region encompassing more than 30% of the chromosome’s total length is regarded as a potential reproductive risk, with clinical monitoring especially recommended when the inverted segment exceeds 50% of the chromosome (Morel et al., 2007; Martin et al., 1994).

Preimplantation genetic testing (PGT) is widely used in couples with balanced chromosomal rearrangements to enhance pregnancy outcomes and shorten the time to successful conception (Fischer et al., 2010; Coonen et al., 2020). While reciprocal translocations (RecT) and Robertsonian translocations (RobT) are the primary indications for PGT for structural rearrangements (PGT-SR), there remains no consensus on whether chromosomal inversions should be routinely included. A retrospective analysis of 576 blastocysts from 57 paracentric and 94 pericentric inversion carriers revealed that the likelihood of unbalanced rearrangements depends on the relative size of the inverted segment in both inversion types and is also influenced by the sex of the carrier (Xie et al., 2019). In contrast, another study involving 283 blastocysts from 57 inversion carrier couples found that embryo ploidy status was not significantly affected by the type of inversion (Tong et al., 2022).

This study aims to analyze and compare the euploid rates of blastocysts and pregnancy outcomes in couples with chromosomal inversions who have undergone PGT, in order to provide subgroup-specific genetic counseling and optimize clinical recommendations.

## Methods

This study retrospectively included couples who received PGT at the Department of Medical Genetics Center, West China Second University Hospital, Sichuan University, between January 2019 and December 2024. Inclusion criteria are couples with complete clinical data, and undergoing PGT due to balanced chromosome rearrangement or recurrent miscarriage. We excluded couples in which the female partner was older than 38 years to minimize the potential confounding effect of advanced maternal age. Ethical approval was granted by the Institutional Review Board (IRB) of West China Second University Hospital, Sichuan University.

Basic information of female age, male age, peripheral blood karyotype results, and reproductive history prior to PGT, including miscarriages (spontaneous abortions), induced abortions (medically indicated terminations due to identified fetal anomalies or chromosomal abnormalities), and live births, was collected. Intracytoplasmic sperm injection (ICSI) was performed to achieve fertilization. Morphological grading of the blastocyst was assessed and classified into stages 3–6 based on inner cell mass, trophectoderm quality, and expansion level. On day 5 or 6 post-fertilization, for blastocyst morphology higher than 4BC, mechanical zona pellucida opening was performed, followed by trophectoderm biopsy of 5–8 cells from each blastocyst.

The biopsied cells were immediately placed in G-MOPS PLUS biopsy medium and then carefully aspirated into sample preservation tubes containing lysis buffer using calibrated pipettes with sterile tips. Biopsied blastocysts were immediately cryopreserved using vitrification. Biopsied samples were subjected to whole genome amplification (WGA) using the MALBAC WGA kit (Yikon Genomics). The amplified products were subsequently used to construct an NGS library with an NGS library preparation kit (Yikon Genomics) according to the manufacturer’s instructions. Sequencing was performed on the NextSeq CN500 platform (Illumina). Raw data were converted to FASTQ files and analyzed using ChromGo software (Yikon Genomics). Quality control criteria required valid reads with GC content between 42% and 47% and a coefficient of variation (CV) below 0.15. For chromosomal copy number variation analyses, a minimum of 1.0 million unique mapping reads (UniqueReads) was required to achieve 4 Mb resolution; for PGT-SR cases involving smaller translocation fragments, a minimum of 3.0 million UniqueReads was required to achieve 1 Mb resolution. The data were analyzed in parallel by two qualified laboratory technicians. Chromosome trisomies, monosomies, deletions or duplications exceeding 4 Mb, structural abnormalities involving rearranged chromosomes larger than 1 Mb, and chromosomal fragments greater than 10 Mb, all at a chimerism level of ≥30% were reported as aneuploidy. Aneuploid cases were further classified as rearranged or non-rearranged chromosome-associated abnormalities.

In couples with a transferable embryo, endometrial preparation was performed using standardized hormone replacement therapy (HRT) protocols. A single embryo transfer was performed only when endometrial thickness was ≥5 mm. Pregnancy outcomes were followed up on and collected. Live birth was defined as pregnancies continuing beyond 28 gestational weeks, implantation failure refers to a negative hCG result after embryo transfer, and pregnancy loss encompassed biochemical pregnancy, ectopic pregnancy, and miscarriage. Prenatal diagnosis was primarily performed via amniocentesis between 18 and 22 weeks of gestation.

All participating couples were grouped according to karyotype findings: PGT-INV (chromosomal inversion), PGT-PV (chromosomal polymorphic variation), PGT-A (normal karyotype), and PGT-SR (other balanced chromosomal rearrangements). Individuals carrying inversions classified as chromosomal polymorphisms—specifically inv(9)(p12q13) and inv(Y)(p11q11)—were assigned to the PGT-PV group. The PGT-INV group was further stratified into subgroups based on inversion type (pericentric vs. paracentric), carrier gender (male vs. female), and the ratio of inverted segment length to total chromosome length is estimated visually from G-banded karyograms (Supplementary File 1): LEVEL 1 (0%–25%), LEVEL 2 (25%–50%), LEVEL 3 (50%–75%), and LEVEL 4 (75%–100%).

SPSS software (version 27.0.1; IBM, Armonk, NY, USA) was used for data analysis. For continuous variables that did not follow a normal distribution, data were summarized using medians and interquartile ranges, and group comparisons were performed using the Kruskal–Wallis test. Categorical variables were presented as proportions (%). The Fisher’s exact test or chi-square test was applied to the comparison among groups. Post-hoc pairwise comparisons with Bonferroni-corrected *P*-values were used to further analyze between-group differences when significant differences were observed.

The Generalized Estimating Equations (GEE) were employed with an exchangeable working correlation matrix to account for the clustering effect within couples. Covariates, including female’s age, history of recurrent miscarriage, embryo development days, expansion degree, and embryo grading, were incorporated to ensure the results are both scientific and accurate. Statistical significance was defined as a two-tailed *P*-value <0.05.

## Results

### Basic information

We included clinical data from 442 blastocysts of 92 couples with chromosomal inversion in the PGT-INV group, 68 blastocysts from 15 couples with chromosomal polymorphic variations in the PGT-PV group, 2,531 blastocysts from 582 couples with normal karyotypes in the PGT-A group, and 5,927 blastocysts from 1,081 couples in the PGT-SR group. Regarding baseline characteristics, female age, male age, and the proportion of prior miscarriages before PGT were significantly higher in the PGT-PV and PGT-A groups than in the PGT-INV and PGT-SR groups (*P* < 0.001). The rate of prior miscarriage before PGT was significantly higher in couples with RecT than those with RobT (*P* < 0.05). No statistically significant differences were observed among the four groups in the rates of induced abortion prior to PGT or live birth prior to PGT (*P* > 0.05). Although the live birth rate before PGT was comparable between RecT and RobT couples undergoing PGT cycles, the rate of induced abortion prior to PGT was significantly higher in RobT couples than in RecT couples (*P* < 0.05) (Table 1). Detailed karyotype findings for the PGT-INV and PGT-PV groups are provided in Supplementary Table 1.

**TABLE 1:** Basic information of included couples underwent PGT.

Category	N	Female age (years)	Male age (years)	Miscarriage prior to PGT	Induced abortion prior to PGT	Live birth prior to PGT
PGT-INV	92	31 (28, 34)	31.5 (29, 34)	57.61% (53/92)^c^	3.26% (3/92)	6.52% (6/92)
Pericentric	47	31 (29, 33)	32 (29, 34)	72.34% (34/47)	6.38% (3/47)	8.51% (4/47)
Paracentric	45	30 (27, 34)	31 (28.25, 35.75)	42.22% (19/45)	0.00% (0/45)	4.44% (2/45)
PGT-PV	15	34 (32, 35)	36 (32.5, 39)	80.00% (12/15)	0.00% (0/15)	0.00% (0/15)
PGT-A	582	33 (31, 35)	34 (32, 37)	91.07% (530/582)	6.01% (35/582)	6.70% (39/582)
PGT-SR	1081	30 (28, 32)	31 (29, 34)	60.59% (655/1081)^c^	4.81% (52/1081)^c^	4.44% (48/1081)
RecT	809	30 (28, 32)	31 (29, 34)	64.40% (521/809)	3.71% (30/809)	4.20% (34/809)
RobT	272	31 (28, 33)	32 (30, 35)	49.26% (134/272)	8.09% (22/272)	5.15% (14/272)
*P*		0.000^a^	0.000^a^	0.000^b^	0.317	0.102

Female age and male age were presented as Median (interquartile range). Miscarriage, spontaneous abortion, and live birth prior to PGT were reported as percentage (frequency).

N, number of couples; PGT, preimplantation genetic testing; PGT-INV, PGT for chromosomal inversion; PGT-PV, PGT for chromosomal polymorphic variation; PGT-A, PGT for aneuploidy in couples with normal karyotype; PGT-SR, PGT for couples with balanced chromosomal rearrangements excepting inversion; RecT, reciprocal translocation; RobT, Robertsonian translcation.

^a^ No significant differences were observed between PGT-A and PGT-PV, whereas significant differences were detected across the other group comparisons.

^b^ Significant differences were observed between the PGT-A and PGT-INV groups, as well as between the PGT-A and PGT-SR groups.

^c^ Significant differences were observed between the subgroups.

### Euploid rate of blastocysts

GEE analysis showed that the euploid rate of biopsied blastocysts did not differ significantly between the PGT-INV and PGT-PV groups (54.30% vs. 66.18%) or between the PGT-INV and PGT-A groups (54.30% vs. 58.63%) (*P* > 0.05). However, the euploid rate in the PGT-SR group (33.24%) was significantly lower than in the other three groups (*P* = 0.000). When considering aneuploid blastocysts with rearrangement-associated chromosomal abnormalities, the proportion in the PGT-INV group (32.18%) was significantly lower than that in the PGT-SR group (75.56%, adj. OR = 6.778, 95% CI: 4.754–9.666, *P* = 0.000) (Table 2; Supplementary Table 2).

**TABLE 2:** Genetic testing results and clinical outcomes of the included blastocysts.

Category	Euploid	Aneuploid			Live birth	Implantation failure	Pregnancy loss^a^	Prenatal diagnosis
		Total	Rearranged chromosome associated	Non-rearranged chromosome associated				
PGT-INV	54.30% (240/442)	45.70% (202/442)	32.18% (65/202)	67.82% (137/202)	54.46% (55/101)	20.79% (21/101)	24.75% (25/101)	36.36% (20/55)
PGT-PV	66.18% (45/68)	33.82% (23/68)	0 (0/23)	100% (23/23)	47.06% (8/17)	23.53% (4/17)	29.41% (5/17)	12.50% (1/8)
PGT-A	58.63% (1484/2531)	41.37% (1047/2531)	—	—	47.95% (292/609)	34.65% (211/609)	17.40% (106/609)	4.45% (13/292)
PGT-SR	33.24% (1970/5927)^d^	66.76% (3957/5927)^d^	75.56% (2990/3957)^d^	24.44% (967/3957)^d^	55.41% (599/1081)	25.25% (273/1081)	19.34% (209/1081)	34.72% (208/599)
RecT	29.17% (1317/4515)	70.83% (3198/4515)	80.08% (2561/3198)	19.92% (637/3198)	56.13% (430/766)	25.46% (195/766)	18.41% (141/766)	36.51% (157/430)
RobT	46.25% (653/1412)	53.75% (759/1412)	56.52% (429/759)	43.48% (330/759)	53.65% (169/315)	24.76% (78/315)	21.59% (68/315)	30.18% (51/169)
*P*	<0.05^b^		<0.05^c^		<0.05^e^ ^,^ ^f^	<0.05^e^ ^,^ ^g^	<0.05^e^ ^,^ ^h^	<0.05^i^

PGT, preimplantation genetic testing; PGT-INV, PGT, for chromosomal inversion; PGT-PV, PGT, for chromosomal polymorphic variation; PGT-A, PGT, for aneuploidy in couples with normal karyotype; PGT-SR, PGT, for couples with balanced chromosomal rearrangements excepting inversion; RecT, reciprocal translocation; RobT, robertsonian translcation.

^a^ Biochemical pregnancy, ectopic pregnancy, and miscarriage were included.

^b^ *P*-value was calculated using GEE, to account for within-couple correlation, with maternal age included as covariate to adjust for potential confounding. Details are provided in Supplementary Table 2. The euploid rate in the PGT-SR, group were significantly lower than the other three groups, and aneuploid rate in the PGT-SR, group were significantly higher than the other three groups.

^c^ The rearranged chromosome associated aneuploidy rate in the PGT-SR, group is significantly higher than those in PGT-INV, group.

^d^ Significant differences were observed between the subgroups.

^e^ *P*-value was calculated using GEE, to account for within-couple correlation, with developmental day, expansion degree, embryo grading, maternal age, and history of recurrent miscarriage included as covariates to adjust for potential confounding. Details are provided in Supplementary Table 2.

^f^ Significant differences were observed between PGT-A, and PGT-SR, groups.

^g^ Significant differences were observed between PGT-INV, group and PGT-A, group (adj. OR, 0.518, 95% CI: 0.293–0.915,*P* = 0.024.).

^h^ Significant differences were observed between PGT-INV, group and PGT-SR, group (adj. OR, 0.150, 95% CI: 0.097–0.232, *P* = 0.000.), PGT-INV, group and PGT-A, group (adj. OR, 0.328, 95% CI: 0.111–0.971, *P* = 0.044), as well as between PGT-A, group and PGT-PV, group (adj. OR, 2.160, 95% CI: 1.045–4.467, *P* = 0.038).

^i^ Significant differences were observed between PGT-A, and PGT-SR, groups (χ2 = 96.453, *P* < 0.001), and between PGT-INV, and PGT-A, groups (χ2 = 54.768, *P* < 0.001).

### Pregnancy outcomes after embryo transfer

GEE analysis showed that the live birth rate (55.41%) in the PGT-SR group was slightly higher than that of the PGT-A group (47.95%) (*P* = 0.049), and comparable to the PGT-INV (54.46%) or PGT-PV (47.06%) (*P* > 0.05), despite the PGT-SR group exhibiting the lowest euploid rate among the four groups. In addition, the implantation failure rate in the PGT-INV group (20.79%) was significantly lower than that in the PGT-A group (34.65%) (P = 0.024), whereas it was comparable to those in the PGT-PV (23.53%) and PGT-SR (25.25%) groups (P > 0.05). Additionally, regarding pregnancy loss—including biochemical pregnancy, ectopic pregnancy, and miscarriage, significant differences were observed between PGT-INV and PGT-SR groups (P = 0.000), PGT-A and PGT-INV groups (P = 0.044), and between PGT-A and PGT-PV groups (P = 0.038). The prenatal diagnosis rate was highest in the PGT-SR and PGT-INV groups (34.72% and 36.36%, respectively), followed by the PGT-PV group (12.5%), and lowest in the PGT-A group (4.45%) (*P* = 0.000). All prenatal diagnosis results were consistent with the embryo’s genetic testing findings (Table 2; Supplementary Table 2).

### Morphology rating of biopsied blastocyst

**FIGURE 1:**
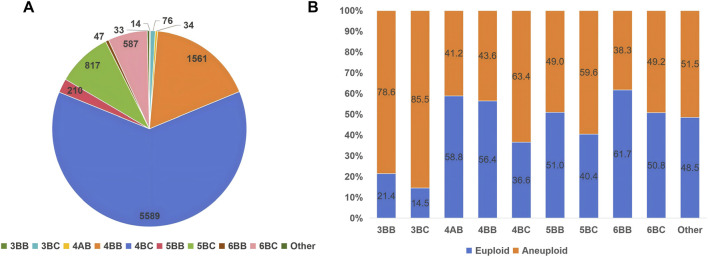
Morphology assessment of biopsied blastocyst **(A)** Distribution of blastocysts across different morphology grades. **(B)** Euploid and aneuploid rates associated with each morphological grade.

Additionally, a possible correlation was observed between the morphological grading of the included blastocysts and their genetic testing results. Among the cohort, 5589 blastocysts were graded with 4BC, followed by 1561 with 4BB, 817 with 5BC, 587 with 6BC, 210 with 5BB, 76 with 3BC, 47 with 6BB, 34 with 4AB, 14 with 3BB and 33 graded under other grades. The highest euploid rate was observed in blastocysts with a 6BB grade (61.7%), followed by 4AB (58.8%) and 4BB (56.4%), whereas the lowest euploid rate was found in the 3BC group (14.5%) (Figure 1).

### Subgroup analysis of the PGT-INV group

Couples with PGT-INV were further stratified by carrier gender, inversion type, and size. The latter was defined as the ratio of the inverted fragment length to the total chromosome length. Results showed that blastocysts derived from male inversion couples demonstrated a significantly higher euploidy rate than those from female inversion couples (61.06% vs. 48.29%; *P* = 0.046). In contrast, the proportion of aneuploidy affecting rearranged chromosomes did not differ significantly between the two subgroups (28.40% for males vs. 34.71% for females; *P* = 0.503). Furthermore, no statistically significant differences were found between male and female carriers in live birth rates (58.70% vs. 50.91%), implantation failure rates (19.57% vs. 21.82%), or pregnancy loss rates (21.74% vs. 27.27%) (all *P* > 0.05) (Table 3).

**TABLE 3:** Comparison of genetic testing results and pregnancy outcomes in subgroup of couples who underwent PGT-INV.

Category	Euploid^a^	Aneuploid^a^			Live birth^b^	Implantation failure^b^	Pregnancy loss^b^ ^,^ ^e^
		Total	Rearranged chromosome associated	Non-rearranged chromosome associated			
PGT-INV	54.30% (240/442)	45.70% (202/442)	32.18% (65/202)	67.82% (137/202)	54.46% (55/101)	20.79% (21/101)	24.75% (25/101)
Male	61.06% (127/208)	38.94% (81/208)	28.40% (23/81)	71.60% (58/81)	58.70% (27/46)	19.57% (9/46)	21.74% (10/46)
Female	48.29% (113/234)	51.71% (121/234)	34.71% (42/121)	65.29% (79/121)	50.91% (28/55)	21.82% (12/55)	27.27% (15/55)
*P*	0.046^c^	​	0.503	​	0.425	0.659	0.929
Pericentric	51.71% (121/234)	48.29% (113/234)	38.94% (44/113)	61.06% (69/113)	56.36% (31/55)	21.82% (12/55)	21.82% (12/55)
Paracentric	57.21% (119/208)	42.79% (89/208)	23.60% (21/89)	76.40% (68/89)	52.17% (24/46)	19.57% (9/46)	28.26% (13/46)
*P*	0.358	​	0.022^d^	​	0.384	0.403	0.570
Level 1	60.16% (77/128)	39.84% (51/128)	11.76% (6/51)	88.24% (45/51)	56.52% (13/23)	21.74% (5/23)	21.74% (5/23)
Level 2	60.00% (87/145)	40.00% (58/145)	25.86% (15/58)	74.14% (43/58)	58.06% (18/31)	12.90% (4/31)	29.03% (9/31)
Level 3	46.94% (46/98)	53.06% (52/98)	44.23% (23/52)	55.77% (29/52)	41.18% (14/34)	32.35% (11/34)	26.47% (9/34)
Level 4	42.25% (30/71)	57.75% (41/71)	51.22% (21/41)	48.78% (20/41)	76.92% (10/13)	7.69% (1/13)	15.38% (2/13)
*P*	>0.05		<0.05^f^		<0.05^g^	>0.05	>0.05
Score; *P*	8.450; 0.004		20.415; 0.000		0.030; 0.862	0.006; 0.939	0.075; 0.785

^a^ *P*-values were calculated using GEE, to account for within-couple correlation, with maternal age included as covariate to adjust for potential confounding.

^b^ *P*-values were calculated using GEE, to account for within-couple correlation, with developmental day, expansion degree, embryo grading, maternal age, and history of recurrent miscarriage included in the model as categorical covariates to adjust for potential confounding.

^c^ Adj. OR, 1.637, 95% CI: 1.008–2.657.

^d^ Adj. OR, 2.228, 95% CI: 1.120–4.432.

^e^ Biochemical pregnancy, ectopic pregnancy, and miscarriage were included.

^f^ Significant differences were observed between LEVEL1 and LEVEL4 (adj. OR, 7.379, 95% CI: 3.016–18.051, *P* = 0.000), as well as between LEVEL2 and LEVEL4 (adj. OR, 2.563, 95% CI: 1.204–5.456, *P* = 0.015).

^g^ With LEVEL 4 as the reference, GEE, analysis indicated significant differences for the other three levels (ORs ranging from 0.071 to 0.116).

With regard to inversion subtypes—pericentric versus paracentric—no statistically significant differences were detected in euploidy rates (51.71% vs. 57.21%; *P* = 0.358) or in clinical outcomes, including live birth (56.36% vs. 52.17%), implantation failure (21.82% vs. 19.57%), and pregnancy loss (21.82% vs. 28.26%) (*P* > 0.05). However, the aneuploidy rate associated with rearranged chromosomes was significantly higher in the pericentric subgroup than in the paracentric subgroup (38.94% vs. 23.60%; *P* = 0.022) (Table 3).

When PGT-INV cases were further categorized by inversion size into four levels—LEVEL 1 (0%–25%), LEVEL 2 (25%–50%), LEVEL 3 (50%–75%), and LEVEL 4 (75%–100%)–a progressive increase in both the overall aneuploidy rate and the proportion of aneuploid blastocysts attributable to homologous recombination was observed, demonstrating a clear linear trend (*P* < 0.05). GEE analysis showed that significant differences in the aneuploidy rate associated with rearranged chromosomes and live birth rates were observed across the four levels (*P* < 0.05). Furthermore, no statistically significant differences were observed across the four levels in implantation failure and pregnancy loss (*P* > 0.05) (Table 3).

## Discussion

This study showed that couples with chromosome inversions undergoing PGT achieved a comparable euploid blastocyst rate to that of couples with polymorphic variants or normal karyotypes, and significantly higher than the rate observed in those with translocations. The incidence of rearrangement-associated abnormalities in blastocysts from inversion carriers was markedly lower than in cases involving reciprocal or Robertsonian translocations. Euploid blastocysts derived from inversion carriers had a lower implantation failure rate than those in PGT-A. Moreover, male inversion carriers showed a greater rate of euploid blastocysts compared to female carriers. Pericentric inversion types were associated with a higher likelihood of chromosomal rearrangement-related abnormalities, and a clear linear correlation was observed between inversion size and aneuploidy rates.

Theoretically, most couples undergo karyotype analysis following infertility or recurrent miscarriage. After going through these, they might be willing to pursue proactive prevention to reduce the risk of recurrence. Assisted reproductive technology combined with PGT provides a possible strategy to increase conception rate and reduce spontaneous abortion resulting from embryo chromosomal abnormalities (Fischer et al., 2010; Coonen et al., 2020). It is widely accepted that PGT offers a more rapid pathway to achieve a live birth compared to natural conception, particularly for individuals with translocations who have experienced repeated pregnancy losses and not yet had a live-born child (Otani et al., 2006). However, whether couples with chromosome inversion are indicated for PGT has long been a subject of debate. Individuals may be concerned about the risk of chromosomal abnormalities in their offspring due to inversion. Thus, real-world data on PGT outcomes in couples with chromosome inversion may provide valuable evidence for optimal clinical management.

This retrospective study shows that miscarriage prior to PGT occurs more frequently in cases of pericentric inversion compared to paracentric inversion, which aligns with theoretical expectations (Anton et al., 2005). The aneuploidy rate among biopsied blastocysts from couples with chromosome inversion was approximately 45.70%, which is comparable with those in the PGT-PV and PGT-A. However, in aneuploidy blastocysts, 32.18% of abnormal embryos were attributed to chromosomal rearrangements, which is comparable to the 32.90% reported in a previous study involving 283 blastocysts from 57 couples carrying chromosomal inversions (Tong et al., 2022). Although the proportion of rearrangement-associated abnormalities in the pericentric inversion group was significantly higher than that in the paracentric inversion group within this data set, the overall aneuploidy rates did not differ significantly between the two groups. These findings suggest that pericentric and paracentric inversions may confer comparable reproductive risks, a conclusion partially supported by previous evidence (Shao et al., 2020). Additionally, female carriers exhibited higher rates of aneuploid embryos and a greater proportion of rearrangement-associated chromosome abnormalities than male carriers, a finding consistent with previous reports (Tong et al., 2022) but contradicting other studies (Shao et al., 2020; Mateu-Brull et al., 2019). Accordingly, our results indicate that the carrier’s gender, rather than the inversion type (pericentric or paracentric), should be considered a primary factor in assessing reproductive risk.

Moreover, inversion size also influences the euploid rate and the risk of aneuploidy associated with chromosomal rearrangement—larger inversions are associated with a higher risk of aneuploidy caused by chromosome inversion, indicating the possibility of estimating the risk of chromosomal abnormalities in embryos based on the size of the inverted segment, as meiotic recombination outcomes are influenced by the proportion of the inverted fragment relative to the total chromosome length (Martin et al., 1994; Xie et al., 2019; Zhang et al., 2026). A classification system has been proposed in which inversions involving less than 30% of the chromosome length are associated with no recombinant chromosomes, those between 30% and 50% generate few recombinants, and inversions exceeding 50% are considered clinically significant for genetic risk assessment (Morel et al., 2007). Furthermore, it has been hypothesized that the meiotic behavior of individuals with pericentric inversions is influenced by the morphological characteristics of the affected chromosome, and no increased incidence of aneuploidy unrelated to the inversion, also referred to as the interchromosomal effect, has been identified (Shilova et al., 2025; Young et al., 2019). A more recent study has indicated that carriers of pericentric inversions involving chromosomes 1–3 or 6–12 exhibited an increased likelihood of generating unbalanced embryos (Zhang et al., 2026).

Additionally, our data showed that couples with RecT are more likely to experience spontaneous abortion, whereas those with RobT are more likely to have undergone induced abortion prior to PGT. This difference may be attributed to the specific chromosomes involved in RobT and underscores the need for prenatal diagnosis in couples with chromosomal rearrangements following natural conception (Uehara et al., 1992). Moreover, when transferable embryos are obtained through PGT, the live birth rate in couples with chromosomal RecT or RobT is slightly higher than that in the PGT-A group. This implies that embryo chromosomal abnormalities constitute the main cause of miscarriage or infertility in couples with RecT or RobT, whereas additional underlying factors may contribute to infertility in couples undergoing PGT-A.

Notably, in our study the implantation failure rate was substantially higher in the PGT-A group (34.65%) than in the PGT-INV (20.79%) and PGT-SR (25.25%) groups. The primary indications for PGT-A in this study cohort were recurrent miscarriage (81.43%) and repeated implantation failures (RIF) (11.57%), with couples in which the female partner was older than 38 years explicitly excluded. Notably, the RIF subgroup, characterized by non-embryonic factors affecting implantation, is underrepresented in the PGT-INV group. This clinical distinction may partially account for the disparity in implantation outcomes after PGT between the two groups. Studies have shown that even after transfer of euploid embryos, patients with RIF have significantly lower implantation rates than non-RIF patients because of the impaired endometrial receptivity and other maternal factors (such as immune or thrombotic disorders) (Cozzolino et al., 2020; Dahiphale et al., 2024). By contrast, the PGT-INV group and PGT-SR group is largely composed of carriers of chromosomal inversions. Their infertility causes tend to be relatively simple. Except for the risks related to abnormal meiotic segregation, these individuals have better ovarian reserve and favorable endometrial environment (Ogilvie et al., 2001).

This study also indicated that a positive correlation between blastocyst expansion stage and euploidy rate; higher-stage embryos exhibit higher euploidy rates, a finding consistent with previous studies (Irani et al., 2017). Additional data from well-designed studies are required to further elucidate the further relationship between embryo morphological assessment and chromosomal abnormalities.

Our study has several strengths; we conducted a multifaceted comparison of couples with chromosomal inversions, both relative to other types of chromosomal rearrangements and within subgroups. Our approach enables more precise characterization by accounting for the distinct meiotic behavior of inversions compared to other rearrangements. In contrast to studies that typically classify chromosomal inversions under the broader PGT-SR category.

This study also had limitations, as we pointed out before, usually couples with infertility or miscarriage would be recommended karyotype analysis, thus the real rate of miscarriage experienced by couples with chromosome inversion might be overestimated. To minimize the potential for euploidy associated with advanced maternal age, we included only couples with female age <38 years; thus, the euploid rate in the PGT-A groups might be higher than those reported in the literature. In this cohort, two female carriers of X-chromosome inversions were included in the broader female carrier cohort. Considering that X-chromosome inversions differ fundamentally from autosomal inversions, to assess the robustness of our primary findings, we re-analyzed all primary outcomes after excluding these two cases. The results remained statistically consistent with our original conclusions. Because endometrial thickness was recorded only as a categorical variable rather than a continuous numeric value, individual endometrial thickness measurements were not available for each embryo transfer; consequently, endometrial factors could not be incorporated into the multivariable analysis using the GEE model.

In conclusion, couples with chromosome inversions achieved a euploid blastocyst rate comparable to that of couples with polymorphic variants or normal karyotypes. Female inversion carriers showed a greater rate of aneuploidy blastocysts compared to male carriers, and a clear linear correlation was observed between inversion size and aneuploid rates. The carrier’s gender and inversion size, rather than the inversion type (pericentric or paracentric), should be considered a primary factor in assessing reproductive risk. PGT represents a valuable reproductive option for individuals with large inverted segments and female carriers. More strictly designed large-sample studies are needed to provide more evidence.

## Data Availability

The original contributions presented in the study are included in the article/Supplementary Material, further inquiries can be directed to the corresponding authors.
