# Supplementary material for: Clinical outcomes of preimplantation genetic testing for structural rearrangements in couples with chromosomal inversions: a retrospective analysis
Source: Front Genet. 2026 Apr 24;17:1779551. doi: 10.3389/fgene.2026.1779551 (PMC13153762; doi:10.3389/fgene.2026.1779551)
Supplement: Supplementary file 1 [file Table1.docx]

| \| Karyotypes \| \| \| --- \| --- \| \| 1 \| 46,XY,inv(11)(p15.2q23.3) \| \| 2 \| 46,XX,inv(15)(q15q26.1) \| \| 3 \| 46,XX,inv(18)(p11.1q23) \| \| 4 \| 46,XY,inv(5)(p15.1q13) \| \| 5 \| 46,XX,inv(8)(p23.1q11.23) \| \| 6 \| 46,XX,inv(10)(p15q21) \| \| 7 \| 46,XY,inv(8)(p21.3q22.3) \| \| 8 \| 46,XX,inv(5)(p13.3q35.2) \| \| 9 \| 46,XY,inv(1)(p36.1q41) \| \| 10 \| 46,XX,inv(4)(p15.1q34) \| \| 11 \| 46,XY,inv(4)(p16.1q34) \| \| 12 \| 46,XY,inv(4)(p?15.3q13.1) \| \| 13 \| 46,XY,inv(6)(p21q15) \| \| 14 \| 46,XX,inv(4)(p14q21) \| \| 15 \| 46,XY,inv(3)(p21.3q25.3) \| \| 16 \| 46,XY,inv(17)(p13q23) \| \| 17 \| 46,XY,inv(20)(p13q13.1) \| \| 18 \| 46,XY,inv(1)(p?34.3q?25.3) \| \| 19 \| 46,XY,inv(3)(p26q25.3) \| \| 20 \| 46,XY,inv(12)(p13.3q21.2) \| \| 21 \| 46,XY,inv(10)(p13q23.2) \| \| 22 \| 46,XX,inv(7)(p14q31.3) \| \| 23 \| 46,XX,inv(4)(p14q27) \| \| 24 \| 46,XX,inv(5)(p15.3q23.3) \| \| 25 \| 46,XY,inv(8)(p11.2q24.3?) \| \| 26 \| 46,XY,inv(7)(p13q31.3) \| \| 27 \| 46,XX,inv(7)(p21q21.1) \| \| 28 \| 46,XX,inv(2)(p21q31) \| \| 29 \| 46,XX,inv(2)(p23q13) \| \| 30 \| 46,XY,inv(8)(p23.2q22.2) \| \| 31 \| 46,XX,inv(8)(p12q21.3) \| \| 32 \| 46,XY,inv(6)(p21.3q25.3) \| \| 33 \| 46,XY,inv(6)(p24q25.1) \| \| 34 \| 46,XY,inv(6)(p21.2q22.3) \| \| 35 \| 46,XY,inv(11)(p11.2q14) \| \| 36 \| 46,XY,inv(5)(p15.1q35.2) \| \| 37 \| 46,XX,inv(8)(p21q21.1) \| \| 38 \| 46,XX,inv(1)(p12q12) \| \| 39 \| 46,XX,inv(2)(p23q31) \| \| 40 \| 46,XX,inv(11)(p15q13.1) \| \| 41 \| 46,XX,inv(20)(p13q13.1) \| \| 42 \| 46,XX,inv(5)(p13.3q23.2) \| \| 43 \| 46,XY,inv(7)(p13q22) \| \| 44 \| 46,XX,inv(16)(p11.2q24) \| \| 45 \| 46,XX,inv(3)(p25q29) \| \| 46 \| 46,XY,inv(20)(p13q13.1) \| \| 47 \| 46,XY,inv(10)(p12.2q26.3) \| \| 48 \| 46,XX,inv(1)(p22q11) \| \| 49 \| 46,XX,inv(10)(q11.2q26.2） \| \| 50 \| 46,XX,inv(1)(p31.1p36.1)pat \| \| 51 \| 46,XX,inv(12)(q13q21) \| \| 52 \| 46,XX,inv(3)(q26q21) \| \| 53 \| 46,XX,inv(12)(q12q23) \| \| 54 \| 46,XY,inv(15)(q15q26.1) \| \| 55 \| 46,XY,inv(2)(q?23~31q37.1) \| \| 56 \| 46,XX,inv(2)(p22p11.2） \| \| 57 \| 46,XX,inv(9)(q31q34.2) \| \| 58 \| 46,XY,inv(3)(p25p14.2) \| \| 59 \| 46,XX,inv(7)(p15p22) \| \| 60 \| 46,XX,inv(10)(q11.2q21) \| \| 61 \| 46,XY,inv(8)(p21.3q22.3) \| \| 62 \| 46,XX,inv(15)(q15q24) \| \| 63 \| 46,XY,inv(5)(q31.1q35.1) \| \| 64 \| 46,XX,inv(20)(p12p11.2) \| \| 65 \| 46,XX,inv(8)(p23.1p21.3) \| \| 66 \| 46,XX,inv(8)(p23p21) \| \| 67 \| 46,X,inv(X)(p11.4p22.31) \| \| 68 \| 46,XX,inv(5)(q31.1q35.1) \| \| 69 \| 46,XX,inv(8)(p23.1p21.2) \| \| 70 \| 46,XY,inv(7)(p22p13) \| \| 71 \| 46,X,inv(X)(p22.p11.2) \| \| 72 \| 46,XX,inv(8)(p23.1p21.2) \| \| 73 \| 46,XY,inv(10)(q21.3q24.1) \| \| 74 \| 46,XX,inv(11)(p15p13) \| \| 75 \| 46,XY,inv(7)(p22p21) \| \| 76 \| 46,XY,inv(8)(p21p11) \| \| 77 \| 46,XY,inv(3)(q25q26.2) \| \| 78 \| 46,XX,inv(13)(q12.3q32) \| \| 79 \| 46,XY,inv(5)(q31.1q34) \| \| 80 \| 46,XY,inv(13)(q33q34) \| \| 81 \| 46,XY,inv(7)(p21p12） \| \| 82 \| 46,XX,inv(2)(q31q32) \| \| 83 \| 46,XY,inv(1)(q25.3q44) \| \| 84 \| 46,XX,inv(12)(q13.1q22) \| \| 85 \| 46,XY,inv(9)(q22q34) \| \| 86 \| 46,XX,inv(2)(p15p11.2) \| \| 87 \| 46,XX,inv(1)(q32q42) \| \| 88 \| 46,XX,inv(6)(p22.2p11.2) \| \| 89 \| 46,XY,inv(2)(p24p23) \| \| 90 \| 46,XX,inv(12)(q13.2q23) \| \| 91 \| 46,XY,inv(5)(q11.2q15) \| \| 92 \| 46,XX,inv(9)(q21q22) \| \| 93 \| 46,XX,inv(9)(p12q13) \| \| 94 \| 46,XX,inv(9)(p12q13) \| \| 95 \| 46,XX,inv(9)(p12q13) \| \| 96 \| 46,XX,inv(9)(p12q13) \| \| 97 \| 46,XX,inv(9)(p12q13) \| \| 98 \| 46,XY,inv(9)(p12q13) \| \| 99 \| 46,XX,inv(9)(p12q13) \| \| 100 \| 46,XY,inv(Y)(p11.2q11.2) \| \| 101 \| 46,XX,inv(9)(p11q13) \| \| 102 \| 46,XX,inv(9)(p11q13) \| \| 103 \| 46,XY,inv(9)(p12q13) \| \| 104 \| 46,XY,inv(Y)(p11q11) \| \| 105 \| 46,XX,inv(9)(p12q13) \| \| 106 \| 46,XX,inv(9)(p12q13) \| \| 107 \| 46,XY,inv(9)(p12q13) \| |
| --- | --- | --- | --- | --- | --- | --- | --- | --- | --- | --- | --- | --- | --- | --- | --- | --- | --- | --- | --- | --- | --- | --- | --- | --- | --- | --- | --- | --- | --- | --- | --- | --- | --- | --- | --- | --- | --- | --- | --- | --- | --- | --- | --- | --- | --- | --- | --- | --- | --- | --- | --- | --- | --- | --- | --- | --- | --- | --- | --- | --- | --- | --- | --- | --- | --- | --- | --- | --- | --- | --- | --- | --- | --- | --- | --- | --- | --- | --- | --- | --- | --- | --- | --- | --- | --- | --- | --- | --- | --- | --- | --- | --- | --- | --- | --- | --- | --- | --- | --- | --- | --- | --- | --- | --- | --- | --- | --- | --- | --- | --- | --- | --- | --- | --- | --- | --- | --- | --- | --- | --- | --- | --- | --- | --- | --- | --- | --- | --- | --- | --- | --- | --- | --- | --- | --- | --- | --- | --- | --- | --- | --- | --- | --- | --- | --- | --- | --- | --- | --- | --- | --- | --- | --- | --- | --- | --- | --- | --- | --- | --- | --- | --- | --- | --- | --- | --- | --- | --- | --- | --- | --- | --- | --- | --- | --- | --- | --- | --- | --- | --- | --- | --- | --- | --- | --- | --- | --- | --- | --- | --- | --- | --- | --- | --- | --- | --- | --- | --- | --- | --- | --- | --- | --- | --- | --- | --- | --- | --- | --- | --- | --- | --- | --- | --- | --- | --- |
| Autosomal Inversion (n=90)  X-Chromosome Inversion (n=2) |
