# Supplementary figures and images for: Clinical outcomes of preimplantation genetic testing for structural rearrangements in couples with chromosomal inversions: a retrospective analysis
Source: Front Genet. 2026 Apr 24;17:1779551. doi: 10.3389/fgene.2026.1779551 (PMC13153762; doi:10.3389/fgene.2026.1779551)

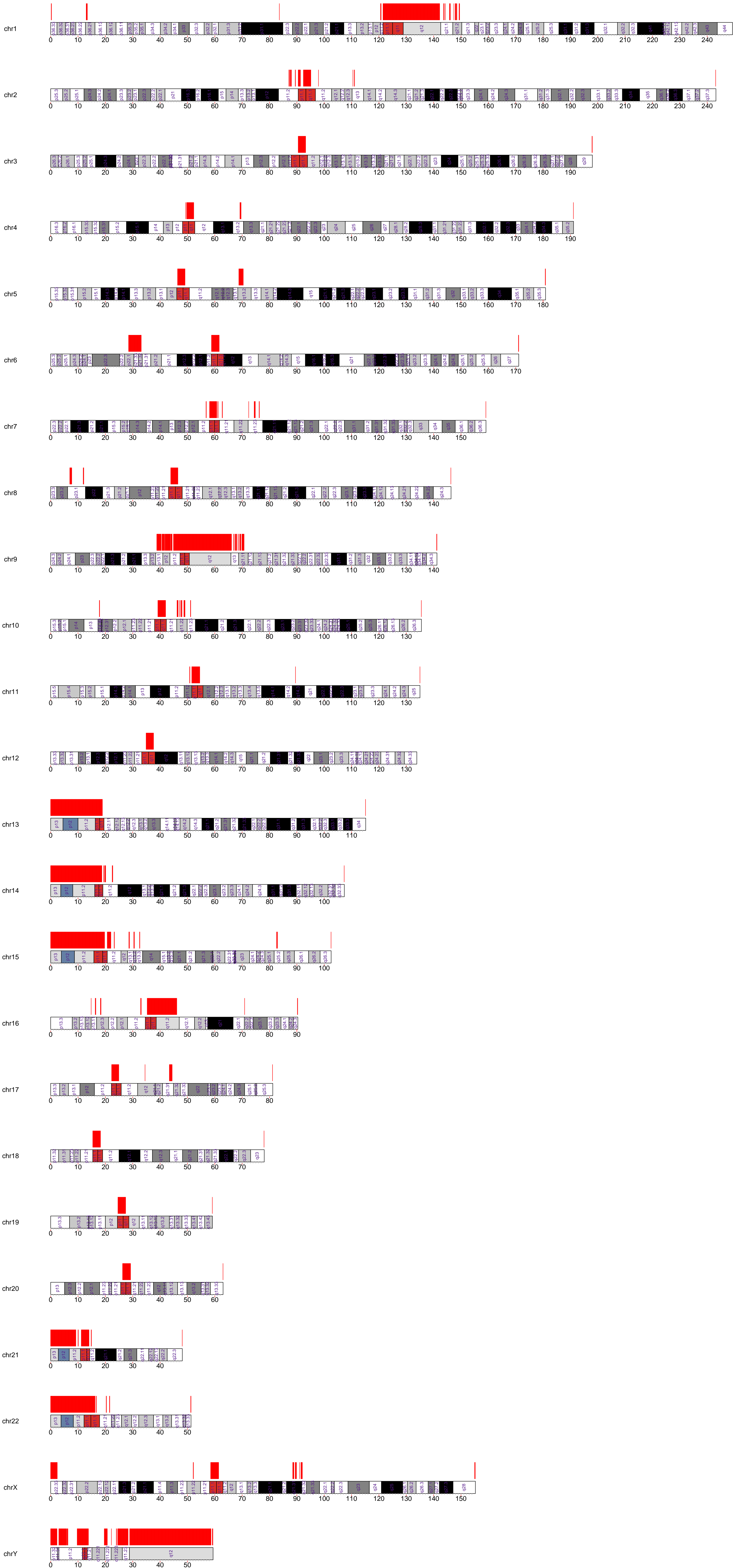

Supplement: Supplementary file 2 [file Supplementaryfile1.pdf]
