# Supplementary material for: Clinical outcomes of preimplantation genetic testing for structural rearrangements in couples with chromosomal inversions: a retrospective analysis
Source: Front Genet. 2026 Apr 24;17:1779551. doi: 10.3389/fgene.2026.1779551 (PMC13153762; doi:10.3389/fgene.2026.1779551)
Supplement: Supplementary file 3 [file Table2.docx]

**Supplementary Table 2-1 Euploid** **of the included blastocysts**

|  | | **Adj. OR** | **95% CI** | **P** |
| --- | --- | --- | --- | --- |
| **PGT-INV** | | 2.390 | 1.871-3.053 | 0.000 |
| **PGT-A** | | 3.104 | 2.76-3.485 | 0.000 |
| **PGT-PV** | | 4.131 | 2.304-7.48 | 0.000 |
| **PGT-SR(Ref)** | | 1 | - | - |
| **Female Age** | | | | |
|  | **<30** | 1.500 | 1.241-1.812 | 0.000 |
|  | **31-35** | 1.342 | 1.119-1.610 | 0.002 |
|  | **36-37(Ref)** | 1 | - | - |

*P*-value was calculated using GEE to account for within-couple correlation, with maternal age included as covariate to adjust for potential confounding.

**Supplementary Table 2-2 Live birth of the included blastocysts**

|  | | **Adj. OR** | **95% CI** | **P** |
| --- | --- | --- | --- | --- |
| **PGT-INV** | | 1.706 | 0.979-2.971 | 0.059 |
| **PGT-A** | | 0.638 | 0.408-0.998 | 0.049 |
| **PGT-PV** | | 3.489 | 0.454-26.822 | 0.230 |
| **PGT-SR(Ref)** | | 1 | - | - |
| **Development Days** | | | | |
|  | **5** | 1.179 | 0.897-1.550 | 0.239 |
|  | **6** | 1 | - | - |
| **Grade** | | | | |
|  | **BB** | 1.726 | 1.203-2.476 | 0.003 |
|  | **BC** | 1 | - | - |
| **Expansion** | | | | |
|  | **4** | 0.552 | 0.298-1.022 | 0.059 |
|  | **5** | 0.929 | 0.543-1.591 | 0.790 |
|  | **6** | 1 | - | - |
| **Miscarriage** | | | | |
|  | **<2** | 1.178 | 0.831-1.671 | 0.358 |
|  | **≥2** | 1 | - | - |
| **Female Age** | | | | |
|  | **<30** | 1.583 | 0.926-2.706 | 0.093 |
|  | **31-35** | 1.370 | 0.896-2.094 | 0.896 |
|  | **36-37** | 1 | - | - |

1. value was calculated using GEE to account for within-couple correlation, with developmental day, expansion degree, embryo grading, maternal age, and history of recurrent miscarriage included as covariates to adjust for potential confounding. To guarantee statistical reliability, embryos with grades 4BA (n=1), 5BA (n=1), and 4AB (n=9) were merged with the 4BB group, collectively defining the cohort of high-quality embryos with high implantation potential.

**Supplementary Table 2-3 Clinical outcomes of the included blastocysts**

|  | | Implantation failure | | | Pregnancy loss | | |
| --- | --- | --- | --- | --- | --- | --- | --- |
|  | | **Adj. OR** | **95% CI** | **P** | **Adj. OR** | **95% CI** | **P** |
| **PGT-INV** | | 0.750 | 0.457-1.231 | 0.256 | 0.150 | 0.097-0.232 | 0.000 |
| **PGT-A** | | 1.305 | 0.978-1.743 | 0.071 | 1.082 | 0.815-1.437 | 0.586 |
| **PGT-PV** | | 0.773 | 0.298-2.055 | 0.597 | 0.551 | 0.253-1.203 | 0.135 |
| **PGT-SR(Ref)** | | 1 | - | - | 1 | - | - |
| **Development Days** | | | | | | | |
|  | **5** | 1.085 | 0.842-1.399 | 0.527 | 0.780 | 0.586-1.038 | 0.088 |
|  | **6** | 1 | - | - | 1 | - | - |
| **Grade** | | | | | | | |
|  | **BB** | 0.874 | 0.684-1.115 | 0.279 | 1.121 | 0.867-1.450 | 0.384 |
|  | **BC** | 1 | - | - | 1 | - | - |
| **Expansion** | | | | | | | |
|  | **4** | 0.841 | 0.564-1.254 | 0.210 | 1.087 | 0.686-1.723 | 0.722 |
|  | **5** | 0.799 | 0.512-1.246 | 0.322 | 0.908 | 0.543-1.518 | 0.712 |
|  | **6** | 1 | - | - | 1 | - | - |
| **Miscarriage** | | | | | | | |
|  | **<2** | 0.917 | 0.716-1.174 | 0.490 | 0.790 | 0.617-1.012 | 0.062 |
|  | **≥2** | 1 | - | - | 1 | - | - |
| **Female Age** | | | | | | | |
|  | **<30** | 0.657 | 0.437-0.986 | 0.042 | 0.811 | 0.499-1.318 | 0.398 |
|  | **31-35** | 0.908 | 0.622-1.327 | 0.619 | 0.611 | 0.381-0.982 | 0.042 |
|  | **36-37** | 1 | - | - | 1 | - |  |

P-value was calculated using GEE to account for within-couple correlation, with developmental day, expansion degree, embryo grading, maternal age, and history of recurrent miscarriage included as covariates to adjust for potential confounding. To guarantee statistical reliability, embryos with grades 4BA (n=1), 5BA (n=1), and 4AB (n=9) were merged with the 4BB group, collectively defining the cohort of high-quality embryos with high implantation potential.

**Supplementary Table 2-4 Details of the PGT-INV blastocysts**

|  | | **Male** | **Female** | **Pericentric** | **Paracentric** | **Level1** | **Level2** | **Level3** | **Level4** |
| --- | --- | --- | --- | --- | --- | --- | --- | --- | --- |
| **Development Days** | | | | | | | | | |
|  | **5** | 18.10%  (80/442) | 20.59%  (91/442) | 18.55%  (82/442) | 20.14%  (89/442) | 11.99%  (53/442) | 14.03%  (62/442) | 8.60%  (38/442) | 4.07%  (18/442) |
|  | **6** | 28.96%  (128/442) | 32.35%  (143/442) | 34.39%  (152/442) | 26.92%  (119/442) | 16.97%  (75/442) | 18.78%  (83/442) | 14.25%  (63/442) | 11.31%  (50/442) |
| **Grade** | | | | | | | | | |
|  | **AB** | 0%  (0/442) | 0.23%  (1/442) | 0%  (0/442) | 0.23%  (1/442) | 0.23%  (1/442) | 0%  (0/442) | 0%  (0/442) | 0%  (0/442) |
|  | **BB** | 12.67%  (56/442) | 16.74%  (74/442) | 14.03%  (62/442) | 15.38%  (68/442) | 9.95%  (44/442) | 9.50%  (42/442) | 7.01%  (31/442) | 2.94%  (13/442) |
|  | **BC** | 34.39%  (152/442) | 35.97%  (159/442) | 38.91%  (172/442) | 31.45%  (139/442) | 18.78%  (83/442) | 23.30%  (103/442) | 15.83%  (70/442) | 12.44%  (55/442) |
| **Expansion** | | | | | | | | | |
|  | **3** | 0.23%  (1/442) | 0.45%  (2/442) | 0.23%  (1/442) | 0.45%  (2/442) | 0%  (0/442) | 0.23%  (1/442) | 0.23%  (1/442) | 0.23%  (1/442) |
|  | **4** | 37.56%  (166/442) | 43.21%  (191/442) | 44.12%  (195/442) | 36.65%  (162/442) | 22.40%  (99/442) | 27.83%  (123/442) | 17.87%  (79/442) | 12.67%  (56/442) |
|  | **5** | 6.33%  (28/442) | 6.11%  (27/442) | 5.66%  (25/442) | 6.79%  (30/442) | 4.52%  (20/442) | 2.94%  (13/442) | 3.39%  (15/442) | 1.58%  (7/442) |
|  | **6** | 2.94%  (13/442) | 3.17%  (14/442) | 2.94%  (13/442) | 3.17%  (14/442) | 2.04%  (9/442) | 1.81%  (8/442) | 1.36%  (6/442) | 0.90%  (4/442) |
